# Recruiting Complex Medical Patients: Lessons From a Study of Pain in Alzheimer’s Disease and Cancer

**DOI:** 10.1093/geroni/igaf122.586

**Published:** 2025-12-31

**Authors:** Jeffrey Boon, Michelle Crum, Jennifer Moon, Ronald Cowan, Todd Monroe

**Affiliations:** The Ohio State University, Columbus, Ohio, United States; The Ohio State University College of Nursing, Columbus, Ohio, United States; The Ohio State University Comprehensive Cancer Center, Columbus, Ohio, United States; The University of Tennessee Health Science Center Department of Psychiatry, Memphis, Tennessee, United States; The Ohio State University, Columbus, Ohio, United States

## Abstract

We are conducting a study on pain differences among people with Alzheimer’s disease (AD), cancer, and both AD and cancer. We encountered difficulties recruiting a hard to identify group—those with AD and cancer. We initially recruited through newspaper, social media, and public transportation advertisements as well as community health fairs and organizations with low yield. We partnered with clinicians in oncology and dementia-specialty practices but experienced challenges obtaining referrals even with our staff embedded in the clinic. Developing relationships with community centers and retirement communities with memory care facilities supported recruitment of individuals with solely AD or cancer. To reach the AD and cancer group, we finally partnered with cancer clinical trials office recruitment coordinators. With trained recruiters systematically reviewing clinic schedules and proactively approaching individuals meeting screening criteria, our rates of study participants with both AD and cancer increased (0.24/month to 2.26/month). These recruiters are trained for empathy and compassion in recruitment and informed consent. This group also has access to artificial intelligence (AI) chart search tools that are being developed; these tools have not yet been utilized as we explore the ethical and regulatory challenges of AI in recruitment. While our approach included a specialty recruitment service, this proactive clinic screening approach could be modified and replicated by teams in the future to reach difficult to recruit medical populations. We have also theorized reasons for this recruitment difficulty such as concern for comfort and caregiver burden that can be considered as part of future recruitment planning.

